# Advances in molecular adjuvants for nucleic acid vaccines

**DOI:** 10.3389/fimmu.2025.1646800

**Published:** 2025-09-29

**Authors:** Casey E. Hojecki, Nicholas J. Tursi, Cory Livingston, David B. Weiner, Ebony N. Gary

**Affiliations:** ^1^ The Vaccine & Immunotherapy Center, The Wistar Institute, Philadelphia, PA, United States; ^2^ The Perelman School of Medicine, The University of Pennsylvania, Philadelphia, PA, United States

**Keywords:** nucleic acid vaccines, adjuvant, gene-encoded adjuvants, molecular adjuvants, plasmid-encoded, DNA Vaccines, mRNA vaccines

## Abstract

As nucleic acid vaccine technology continues to advance, modern adjuvants are being engineered to quantitatively and qualitatively shape immune responses. Since their development in the early 1990’s, nucleic acid approaches have garnered significant attention, and numerous platform technologies have been developed both to improve delivery as well as immunogenicity. These advances were highlighted during the COVID-19 pandemic, with the approval of both mRNA-LNP and DNA vaccines for SARS-CoV-2. Early clinical trials with DNA antigens alone displayed suboptimal immunogenicity, supporting interest in adjuvant molecules. Molecular adjuvants, nucleic acid-encoded cytokines, chemokines, and enzymes, among others, are used to enhance and direct nucleic acid antigen-induced immunity *in vivo*. Additionally, mRNA-LNP vaccines, and more recently DNA-LNP vaccines, have demonstrated robust immunogenicity with intrinsic adjuvant activity based on the delivery mode. This review summarizes the molecular adjuvant landscape and highlights recent findings in the context of nucleic acid vaccines.

## Introduction

Adjuvants are vaccine components that enhance and direct the immune response. The term comes from the Latin *adjuvare*, meaning to help or aide, and was first used by Gaston Ramon in 1925 after observing that horses with inflammation or abscesses at the site of injection developed higher antibody titers. A year later, the first adjuvant, alum, was discovered serendipitously by Alexander Glenny (1). While attempting to purify diphtheria toxin, he observed that aluminum salts precipitated the toxoid, leading to stronger antibody responses in guinea pigs. The resulting stable, insoluble complexes prolonged antigen exposure to immune cells. Alum was promptly incorporated into human vaccines and remained the only licensed adjuvant for most of the 20th century (1930s-1990s) (2).

Later decades saw growing interest in adjuvants, but alternatives such as Freund’s water-in-oil emulsions developed in the 1940s proved too toxic for human use (1). The next regulatory approvals would not come until the turn of the century, first with MF59, a squalene-based oil-in-water emulsion with surfactants approved in Italy in 1997 for seasonal influenza. AS04 followed in 2005, a combination of monophosphoryl lipid A (MPL) and aluminum salt (aluminum hydroxide) approved in the EU for use in Cervarix, a human papillomavirus (HPV) vaccine. AS03 was approved in 2009 for the H1N1 pandemic, an oil-in-water emulsion containing squalene, DL-α-tocopherol (vitamin E), and polysorbate 80. AS01, a liposome-based adjuvant, was approved in 2017 for the shingles vaccine Shingrix. Finally, CpG 1018, a synthetic 22-mer phosphorothioate-linked oligodeoxynucleotide which acts as a Toll-like receptor 9 (TLR9) agonist, was approved in the US in 2017 for Heplisav B, a hepatitis B vaccine. This string of approvals marked a broader shift from empirical vaccinology to mechanistically-informed adjuvant selection (3) (reviewed by Goetz et al., 2024). These molecules have been used as chemical adjuvants in the context of inactivated virus and protein-based vaccine platforms for decades.

In the nucleic acid platform space, the ability to simultaneously deliver gene-encoded molecular adjuvants to modify vaccine-induced immunity has transformative potential. In 1990, Wolff et al. demonstrated the induced expression of reporter proteins in mouse muscle tissue from RNA and DNA vectors, opening the door for nucleic acid delivery (4). DNA vaccines emerged in the early 1990s, with pivotal work by Weiner and colleagues demonstrating “gene inoculation”, the successful delivery of plasmid DNA to elicit both humoral and cellular responses against HIV-1 env in mice (5). This breakthrough introduced endogenous antigen expression, in which transfected host cells produce the encoded immunogen. The new vaccine platform promised direct immune stimulation of cellular immunity along with traditional antibody responses and the ability to engineer tailored vaccines. Unlike other platforms, this approach reliably enabled antigen processing through the MHC Class I pathway, mimicking viral infection and robustly inducing cytotoxic T lymphocyte (CTL) responses. However, early clinical trials in the late 1990s revealed low expression of the transgene in humans and poor antigen-presenting cell (APC) uptake from naked DNA (6), prompting advancements in delivery technologies and a new era of adjuvant research to enhance immunogenicity.

Electroporation, developed by Inovio and Ichor Medical in the 2000s, uses electrical pulses that transiently permeabilize cell membranes to enhance DNA plasmid uptake, improving immunogenicity in pre-clinical models and humans. Utilizing plasmid DNA as a vector enables co-delivery of gene-encoded adjuvant molecules alongside antigen. Molecular adjuvants can enhance (7–12) and direct (9, 13–17) vaccine-induced immunity *in vivo*, including in clinical trials (18, 19). Through the 2000s, early advancements in DNA vaccine technology included codon optimization (20–22) and exploration into molecular adjuvants, such as IL-12, GM-CSF, and CD40L. Promoter and intron optimizations were also leveraged to boost expression. The COVID-19 pandemic brought rapid development focus to the DNA platform. In 2020, Smith et al. developed a synthetic DNA vaccine, INO-4800, which elicited strong humoral and cellular immune responses in mice and guinea pigs, including neutralizing antibodies and T cells (23). Subsequent Phase 1 and 2 trials demonstrated a favorable safety profile and durable immune responses in humans (24, 25). In 2021, India’s drug regulator approved ZyCoV-D, the world’s first licensed DNA vaccine. Approved for emergency use during the SARS-CoV-2 pandemic, the vaccine delivered plasmid-encoded spike protein via jet injector and demonstrated 66% efficacy in Phase 3 (26). Since the pandemic, clinical development has continued to advance (27–29), with clinical trials for DNA vaccines against HIV-1, HPV, Zika, Ebola, TB (30), and immunotherapies (31, 32). Outside of physical delivery modalities, lipid-based formulations for plasmid DNA vaccines have historically shown limited immunogenicity *in vivo*. However, recent advances in lipid nanoparticle technology, microfluidics, and formulation have improved particle stability and immunogenicity, generating significant interest in LNP-mediated delivery as a viable strategy for the DNA vaccine platform (33–38).

The use of RNA to deliver antigens was initially hindered by instability, rapid degradation, and strong innate immune activation. A major breakthrough was achieved in 2005 when Karikó and Weissman demonstrated that nucleoside modifications including pseudouridine substitution reduced Toll-like receptor activation and enhanced translation efficiency (39). Subsequent innovations included optimized 5′ cap analogs, untranslated regions, and expanded nucleoside chemistries (38, 39). Delivery technologies also progressed, as ionizable lipid nanoparticles (LNPs) evolved from earlier liposome and cationic lipid systems (40). These LNPs were engineered to efficiently encapsulate mRNA, facilitate endosomal escape, and enable cytoplasmic delivery while minimizing toxicity (40) (Reviewed by Hou et al., 2021). By the late 2010s, these advances collectively enabled the first clinical successes in mRNA-based vaccines and therapeutics. In 2018, Alnylam’s *Onpattro*, became the first approved RNA therapeutic (siRNA-LNP) (41) and both Moderna and BioNTech, among other companies advanced clinical-stage mRNA vaccines for Zika, CMV, and certain cancers.

The COVID-19 pandemic potentiated unprecedented levels of development opportunity, accelerating the first widespread use and validation of mRNA vaccine technology. In 2020, mRNA vaccine candidates BNT162b2 (Pfizer-BioNTech) (42) and mRNA-1273 (Moderna) (43) became the first nucleic acid vaccines approved for human use. Key studies have explored the potential of incorporating adjuvant molecules in the mRNA platform, as well as additional formulation, delivery, and sequence-level methods to reduce side effects and address waning immunity (44). Since 2022, the platform has expanded beyond COVID-19 with clinical trials for quadrivalent mRNA influenza vaccines (45), RSV (46, 47), EBV, and renewed efforts against Zika, CMV, and immunotherapies (48, 49). Emerging directions include self-amplifying RNA constructs, thermostable formulations, and tolerogenic vaccines for autoimmune diseases, while personalized cancer vaccines advance into phase 2 and 3 trials (49, 50).

**Figure 1:**
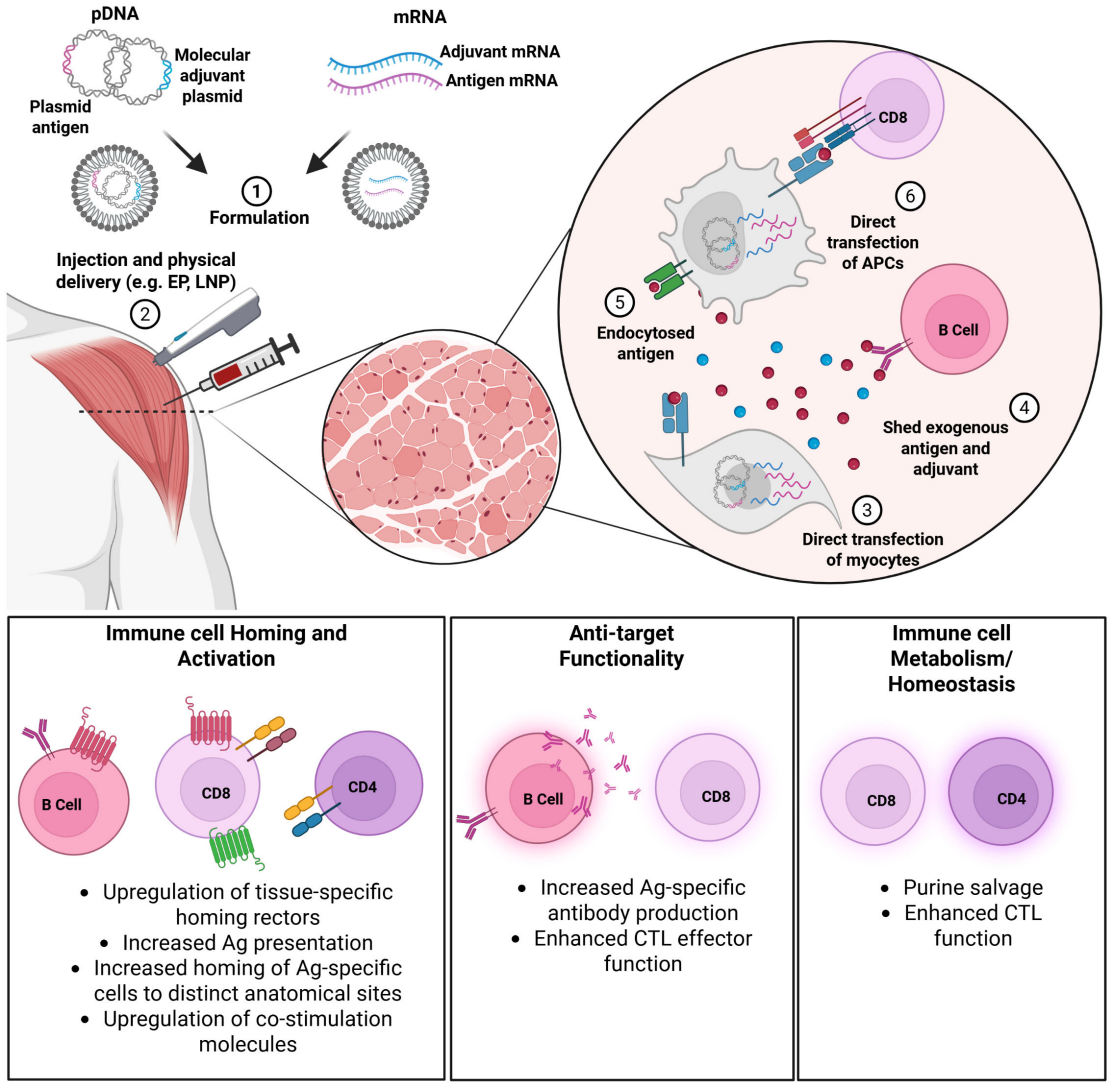
Mechanisms of molecular adjuvant action. Plasmid DNA (pDNA) or messenger RNA (mRNA) molecules encoding the antigen and adjuvant are formulated (1) and delivered (2) by direct injection or other physical delivery methods such as jet, electroporation (EP), or lipid nanoparticle (LNP) encapsulation.

The ability to co-deliver immune-modifying agents alongside the antigenic payload is a key feature of the nucleic acid platform, enabling precise stimulation and tailoring of vaccine-induced immunity *in vivo* (**Figure 1**). In this review, we summarize the history and current landscape of genetic, or molecular adjuvants, with a specific focus on vaccines targeting infectious diseases.

## Cytokine adjuvants

Cytokines are a broad class of molecules involved in intercellular communication and immune regulation, often promoting immune cell proliferation, differentiation, and effector function. Cytokine adjuvants are a subset of these molecules that have demonstrated potential to enhance vaccine-induced immune responses (**Table 1**).

**Table 1:** Molecular adjuvants by class.

Cytokines	Antigens	Significant Findings	Reference
**IL-12**	*Mycobacterium* *tuberculosis (Mtb)*, SARS-CoV-2, *Listeria monocytogenes*-OVA	Sustained immunity to SARS-CoV-2 mRNA-LNP, enhances CTL responses	Morelli et al. (51) Brook et al. (44) Aunins et al. (52)
**IL-2**	rabbit hemorrhagic disease virus VP60, infectious bursal disease virus, *Edwardsiella tarda*, infectious laryngotracheitis virus, autoimmune diabetes	Enhances T cell proliferation and protection across many animal and disease models	Deng et al. (53) Huo et al. (54) Tang et al. (55) Hao et al. (56) Pagni et al. (57)
**IL-4**	Influenza hemagglutinin, coccidiosis	Combination approaches preserve Th1-related responses	Wei et al. (58) Tan et al. (59)
**IL-15**	SIV-Gag/SIV-Nef, HPV16 E6/E7	Enhances CTL responses despite delivery constraints regarding trans-presentation	Leroy et al. (60) Zhou et al. (61)
**IL-18**	genotype VII Newcastle Disease Virus	Enhances Th1-related responses in chickens and murine cancer models	Wang et al. (62) Yadav et al. (63)
**IL-28B** **(IFN-λ3)**	H1N1 (inactivated virus vaccine), Newcastle Disease Virus, HPV16 E6/E7	Promotes robust CD8^+^ activation by T_reg_ suppression	Sabbaghi et al. (64) Amoia et al. (65) Zhou et al. (61)
**GM-CSF**	SARS-CoV-2 WT, Omi, Influenza HA	Enhances germinal center formation	Liu et al. (66) Wei et al. (58)

Chemokines	Antigens	Significant Findings	Reference
**CCR10 Ligands (CCL27, CCL28)**	SARS-CoV-2 WT, BA.2, XBB.1.5, influenza, HIV-1 env	Drive robust mucosal immunity in murine models of challenge	Gary et al. (13) Gary et al. (67) Liaw et al. (68)
**CCR7 Ligands (CCL19, CCL21)**	*Vibrio anguillarum* antigen (VAA), VHSV, H7N9 HA	Enhance systemic & mucosal immunity across animal models	Xu et al. (69) Kim et al. (70) Xiang et al. (71)

Co-stimulators	Antigens	Significant Findings	Reference
**CD40L**	autoimmune glomerulonephritis (EAG), SARS-CoV-2, Tembusu virus, bovine herpesvirus 1 (BoHV-1)	Potent multi-faceted immune enhancement via dendritic cell activation across animal models	Li et al. (72) Tamming et al. (73) Huang et al. (74) Kornuta et al. (75)
**CD80/86**	*Vibrio anguillarum* OmpK	increased IgM+ and CD4+ populations	Liu et al. (76)

Immunomodulators	Antigens	Significant Findings	Reference
**Adenosine deaminase**	HIV-1, SARS-CoV-2	Mitigates age-associated immunosenescence in mouse models	Gary et al. (13) Cusimano et al. (8) Gary et al. (7)
**C3d**	Porcine Circovirus Type 2 (PCV2) ORF2 , SARS-CoV-2	Significantly increases antibody responses	Hou et al. (77) Li et al. (78)

### IL-12

Interleukin-12 (IL-12) is a proinflammatory cytokine that promotes cellular immunity by enhancing CD8^+^ T cell responses, driving Th1 polarization, and stimulating interferon-γ (IFN-γ) production. The first use of IL-12 as an adjuvant dates back to the mid-1990s, with several landmark studies shortly after it was first characterized in 1989 (79) Afonso and colleagues demonstrated recombinant IL-12 to be effective for the induction of cell-mediated immunity against *leishmaniasis* in 1994 (80). Three years later, the first study demonstrating plasmid-encoded IL-12 as a genetic adjuvant was published, with co-delivery generating enhanced cell-mediated immunity for a DNA vaccine encoding several HIV-1 antigens (81). Plasmid-encoded IL-12 has been well-tolerated and shown a significant dose-sparing effect in clinical trials for DNA-based HIV-1 and HCV vaccines (82–85) along with several immunotherapies (32, 86, 87).

Systemic administration of recombinant IL-12 was identified to cause toxicity in both humans and animal models (88), with early clinical trials reporting severe, sometimes fatal effects, including IFNy overproduction and cytokine storm–like responses (89). Subsequent studies confirmed comparable toxicity for IL-12 delivered systemically via naked DNA, however, IL-12 pre-dosing significantly attenuated toxicity, showing a schedule-dependent desensitization effect (88). Since then, strategies have centered on controlling expression through localized delivery, pre-dosing protocols, tunable plasmid-encoded expression vectors, and dosage control.

While IL-12 is a well-characterized cytokine adjuvant, recent studies have applied it in new vaccine contexts, including DNA-encoded delivery for tuberculosis and as a co-adjuvant to improve durability in SARS-CoV-2 mRNA models. A 2020 study demonstrated IL-12 robustly enhanced immune responses in *Mycobacterium tuberculosis (Mtb)* vaccination (51). Prior to this work, IL-12 received limited investigation as an adjuvant for *Mtb* vaccines, hindered by delivery challenges and inconsistent protective outcomes. This study is among the first to demonstrate that IL-12 DNA co-delivery can robustly enhance Ag85A-specific lymphocytes and protection in challenge. In a DNA-A85A/MVA85A prime-boost regimen, IL-12 co-delivery significantly enhanced IFN-γ responses, expanded Ag85A-specific CD4^+^ and CD8^+^ T cells, and increased the cytotoxic CD107a-expressing CD8^+^ T cell population. Morelli et al. also observed increased anti-Ag85A antibody levels and reduced lung bacterial burden post-challenge, indicating improved protection. Importantly no severe adverse events (SAEs) were reported in these trials, further demonstrating the safety of local plasmid delivery of IL-12.

Although SARS-CoV-2 mRNA vaccines induce robust acute immune responses in humans, numerous studies have shown that these responses have limited durability (90). A 2024 study found that IL-12p70-expressing mRNA-LNP could improve response durability (44). Co-delivery improved antibody and cell-mediated immune responses when combined with the Pfizer BNT162b2 SARS-CoV-2 vaccine. In an aged mouse model, the IL-12 mRNA-LNP adjuvant increased humoral immune response durability and spike-specific IgG titers to levels comparable to young adult mice. The IL-12 mRNA-encoded adjuvant was designed with a multiorgan protection (MOP) sequence to restrict expression to the injection site, mitigating toxicity risks. By enhancing immunogenicity in both young and aged mice, this approach demonstrated promise for clinical use in at-risk groups.

Aunins and colleagues further demonstrated the potential of IL-12 as an adjuvant in the mRNA-LNP platform, in a study using models of both bacterial infection and cancer (52). IL-12 mRNA-LNP co-delivery enhanced antigen-specific CD8^+^ T cell expansion, effector function, and memory formation. This methodology led to improved protection in models of *Listeria monocytogenes* infection and B16 melanoma. The IL-12 construct consisted of codon-optimized mRNA encoding the p35 and p40 subunits joined by a flexible glycine-serine linker, enabling co-translation and efficient heterodimerization into functional IL-12p70.

IL-12 is one of the most extensively studied gene-encoded adjuvants, with consistent immunostimulatory effects in preclinical models and an acceptable safety profile in clinical settings. Plasmid-encoded delivery has been central to its development, enabling localized expression that significantly mitigates the systemic toxicity associated with recombinant protein approaches. In DNA-based platforms, p35 and p40 are often expressed from bicistronic plasmids using dual promoters, with staggered strength to favor proper heterodimer assembly, whereas in mRNA vaccines, a single transcript typically encodes both subunits joined by a flexible linker. Recent studies reinforce IL-12’s robust profile as a molecular adjuvant for nucleic acid vaccines, particularly in enhancing Th1-biased cellular immunity. Current efforts focus on refining delivery systems and dosing regimens to enhance efficacy and expand translational development.

### IL-2

Interleukin-2 is a cytokine primarily produced by activated CD4+ T cells that plays a central role in the proliferation, survival, and functional differentiation of T cells and NK cells. IL-2 has long been explored for its ability to enhance vaccine-induced cellular immunity. It was first investigated as an adjuvant in a 1989 study that demonstrated systemic administration of recombinant IL-2 protein alongside an inactivated rabies virus vaccine improved cell-mediated protection in challenge (91). Later studies confirmed co-administration of IL-2 could enhance antigen-specific immune responses, particularly by promoting T cell proliferation and activity. IL-2 was first investigated as a plasmid-encoded adjuvant in 2005, when it was shown murine IL-2 fused to Ig (IL-2/Ig) co-administered with a DNA vaccine encoding HIV-1 env gp120 in mice, significantly enhanced both antibody and cell-mediated immune responses compared to the DNA vaccine alone (92). Recombinant IL-2 has been found to cause significant toxicity from systemic delivery, while plasmid-encoded IL-2 has been well-tolerated in trials for DNA-based cancer therapeutics (93, 94).

Recent findings in IL-2 as an adjuvant have primarily occurred in chicken models, with additional findings in rabbits, mice, and fish. In a 2019 study, researchers developed an oral DNA vaccine using attenuated *Salmonella typhimurium* to deliver a plasmid encoding both IL-2 and the VP60 capsid protein of rabbit hemorrhagic disease virus (RHDV) (53). Co-expression of IL-2 significantly enhanced humoral and cellular immune responses, leading to 93.3% protection against viral challenge, surpassing the efficacy of vaccines lacking IL-2.

A DNA vaccine co-expressing chicken IL-2 (chIL-2) and IL-7 (chIL-7) with the VP2 antigen demonstrated enhanced immunogenicity and protective efficacy against infectious bursal disease (IBDV) in a 2019 study (54). The chIL-2/chIL-7/VP2 combination vaccine significantly increased IBDV VP2-specific antibody titers, T cell proliferation, and IFN-γ production. In 2020 Tang et al. demonstrated that plasmid encoded flounder IL-2 (poIL-2) enhanced protection against *Edwardsiella tarda* (55). Both recombinant (rIL-2) and plasmid-encoded (pcIL-2) forms of poIL-2 significantly improved survival rates, antigen-specific antibody production, and expression of immune-related genes when co-administered with a recombinant OmpV vaccine. However, recombinant IL-2 elicited stronger responses than the plasmid-encoded form.

Similarly, co-delivery of chicken IL-2 (chIL-2) with an infectious laryngotracheitis virus (ILTV) chicken embryo origin (CEO) vaccine significantly alleviated vaccine-induced clinical signs without compromising protective efficacy (56). Oral delivery of chIL-2 reduced viral loads in key respiratory tissues and shortened the duration of adverse reactions. IL-2 enhanced early activation and expansion of natural killer cells and cytotoxic T lymphocytes, particularly in mucosal tissues.

Finally, a 2025 study reported a multi-component DNA-launched plasmid prevented autoimmune diabetes in nonobese diabetic (NOD) mice (57). The construct encoded the cytokines TGF-β1, IL-10, and IL-2 alongside preproinsulin2. IL-2 contributed to antigen-specific immune tolerance without systemic immunosuppression. These findings support the ability of IL-2 to boost cellular immunity across a variety of animal models.

### IL-4

Another well-characterized adjuvant is Interleukin-4, a cytokine that induces differentiation of naive helper T cells (T_H_0 cells) to T_H_2 cells. IL-4 has been noted for its T_H_2 bias and its ability to promote humoral immunity and IgG1/IgE production. Early studies showed that plasmid-encoded IL-4 could enhance antibody responses when co-delivered with DNA vaccines (e.g., for HIV, influenza, or allergens). However, use of IL-4 has been limited due to concerns of skewing away from protective Th1 responses and dampening cell-mediated activity (58, 95).

The earliest study investigating IL-4 as an adjuvant was published in 1998, showing a DNA vaccine developed from an ovalbumin (OVA) and murine IL-4 fusion gene (95). Mice immunized with OVA/IL-4 DNA exhibited enhanced OVA-specific IL-4 production by CD4^+^ T cells and a higher ratio of anti-OVA IgG1 to IgG2a antibodies, indicating a T_H_2-biased response. The OVA/IL4 fusion gene induced T_H_2-biased cell-mediated responses while antigen alone or a mixture of antigen and IL-4 did not, supporting that direct linkage drives the immune response phenotype.

A recent study revisited IL-4 as an adjuvant in a dual-cytokine approach. Wei et al. (58) reported the use of mRNA-encoded GIFT4, a fusion cytokine (fusokine) combining GM-CSF and IL-4. It was originally characterized in 2014 for its ability to drive potent B cell proliferation and high levels of IL-1α, IL-6, IL-12, and IL-5 relative to the combined delivery of recombinant GM-CSF and IL-4 (96). When encoded by mRNA and co-delivered with antigen, GIFT4 enhanced both humoral and cellular responses to influenza in mice, including early germinal center formation and lung-resident T cell populations. The Th2-skewing effect of IL-4 was intentionally leveraged in this strategy, with the GM-CSF and IL-4 fusion selected for its cooperative enhancement of B cell activation and proliferation beyond that of either cytokine alone. This approach focused on improving antibody quality and breadth, rather than quantity alone. The preservation of robust cellular responses suggests that the inclusion of GM-CSF counterbalanced the typical suppressive effects of IL-4 on Th1-related immunity.

Plasmid-encoded IL-4 was implemented in another fusion-based strategy to enhance protection against coccidiosis in chickens (59). The construct pCI-IL-4-IL-2-EGFP, encoding chicken IL-4 and IL-2, significantly boosted both cellular and humoral responses when co-administered with a live coccidia vaccine. This combination increased the expression of IL-2, IL-4, TNF-α, and IFN-γ, and promoted the expansion of B cells, T cells, and APCs in the spleen and intestinal tissues - the primary site of infection. These findings highlight the value of combination approaches for refining the immunomodulatory role of IL-4 to further tailor vaccine-induced immune responses.

### IL-15

Interleukin-15 (IL-15) drives the expansion and survival of memory CD8^+^ T cells and NK cells, which supports its role in promoting cell-mediated immunity. It was first studied as an adjuvant in the late 1990s when recombinant IL-15 was shown to enhance CD8+ T cell responses in a Toxoplasma gondii mouse model (97). The first use of IL-15 as a plasmid-encoded vaccine adjuvant came a decade later. Two key studies from 2005 reported plasmid-encoded IL-15 could enhance cellular immunity when delivered alongside DNA vaccines targeting HIV or herpes simplex virus in mice (98, 99). Plasmid-encoded IL-15 was shown to be well-tolerated in humans but offered no apparent augmentation in a 2012 clinical trial for a HIV gag DNA vaccine (18).

Research on IL-15 has been limited in recent years due to its biological dependence on trans-presentation via IL-15Rα for optimal T cell activation (100–103). Maintaining this complex *in vivo* has proven difficult, shifting focus to alternatives (100). In support of a more feasible nucleic acid based approach, a 2022 study showed that co-delivery of IL-15 as a DNA-encoded adjuvant with a non-integrating lentiDNA SHIV vaccine enhanced vaccine-specific CD4^+^ and CD8^+^ T cell responses in both mice and rhesus macaques (60). IL-15 co-expression also increased the durability of antibody-dependent cellular cytotoxicity (ADCC) responses in plasma and mucosal compartments for up to 40 weeks. In 2025 researchers evaluated IL-15 and IL-28B as a gene-encoded adjuvants for an HPV16 DNA vaccine targeting E6/E7 antigens (61). Co-delivery of IL-15 plasmid enhanced CD8^+^ T cell responses, demonstrated by increased E7-specific IFN-γ secretion relative to antigen alone.

The broader use of IL-15 will likely depend on further improvements to stability and delivery mechanisms to overcome the challenges posed by trans-presentation and half-life constraints (100–104). The successful integration of IL-15 into recent DNA vaccine platforms suggests untapped potential. However, as seen in the HPV16 model, the choice of cytokine must align with the antigen and desired immune profile.

### IL-18

Interleukin-18, initially referred to interferon-γ-inducing factor following its discovery (105) in 1995, is secreted primarily by activated monocytes. IL-18 is best known for promoting T_H_1-related differentiation in the presence of IL-12, though it can also support T_H_2 responses under certain conditions (106). Plasmid-encoded IL-18 has shown the most promise for enhancing cellular immunity in murine cancer models, with many studies in the 2000s (63, 107–110). Species-specific receptor interactions and pro-inflammatory toxicity have limited translation to humans. In contrast, livestock more readily tolerate elevated IFN-γ and permit less stringent formulation constraints. Recent progress in the context of infectious disease has been in veterinary models.

A 2022 study demonstrated that co-delivery of chicken IL-18 significantly enhanced mucosal and systemic immunity against genotype VII Newcastle Disease Virus (62). The cytokine gene was delivered with a minicircle DNA vaccine (pYL58) via attenuated *Salmonella* in chickens. IL-18 co-expression boosted IFN-γ and IFN-α production, improved lymphocyte proliferation, and increased protection post-challenge (70% vs. 50%).

While these findings support the use of IL-18 in veterinary contexts, its efficacy in mammalian models of infectious disease remains underexplored. Future studies should focus on optimizing delivery and resolving its safety profile. Clinical translation of plasmid-encoded IL-18 delivery will require building on the progress in murine cancer models.

### IL-28B

IL-28B, also known as interferon lambda 3 (IFN-λ3), is a member of the type III interferon family that signals through the heterodimeric receptor complex IFNLR1/IL10R2. It plays a critical role in mucosal antiviral defense by inducing interferon-stimulated genes (ISGs) primarily in epithelial and barrier tissues (111). IL-28B is secreted primarily by dendritic cells (DCs) and macrophages and enhances viral clearance by promoting robust CD8^+^ T cell responses while reducing regulatory T cell (T_reg_)-mediated suppression (112). Following the discovery of type III IFNs (IFN-λ or IL-28/29) in 2002, IL-28B’s antiviral and antitumor properties were characterized in multiple disease models (111). Recombinant IL-28 protein completely blocked mucosal replication and disease in an *in vivo* HSV-2 infection model and enhanced systemic IFN-γ responses (111). Plasmid-encoded IL-28B was first evaluated as a vaccine adjuvant in 2009, where co-delivery with an HIV Gag DNA vaccine reduced T_reg_ populations, increased cytotoxic CD8^+^ T cells, and provided full protection in a lethal influenza challenge (112). These findings were extended to non-human primates, with plasmid-encoded IL-28B enhancing antigen-specific CTL responses and cytolytic activity in rhesus macaques vaccinated with plasmid DNA encoding HIV-1 gag and pol (113).

In recent years, IL-28b has been explored as an adjuvant across mice and poultry animal models. A 2021 study reported plasmid-encoded IL-28B co-administered intranasally with a gamma-irradiated H1N1 influenza vaccine, significantly enhanced both mucosal (IgA) and systemic (IgG) antibody responses, as well as T cell proliferation and Th1-related cytokine production (IFN-γ, IL-12) (64). Mice receiving the plasmid-encoded IL-28B adjuvant showed reduced lung viral titers and decreased inflammatory cytokines (IL-6, IL-10) post-challenge, suggesting improved immune regulation.

The inclusion of IL-28b in a genotype-matched Newcastle disease virus DNA vaccine significantly improved immune responses and protective efficacy in chicks (65). The IL-28b-adjuvanted vaccine (pTwist-F-HN-VII-IL28b) induced stronger immunity and achieved 80% protection against a virulent NDV strain, outperforming both the non-adjuvanted plasmid and the conventional LaSota vaccine.

Zhou et al. reported that plasmid-encoded IL-28B significantly enhanced antigen-specific CD8+ T cell responses in a therapeutic HPV16 DNA vaccine targeting E6 and E7 oncoproteins in mice (61). IL-28B was delivered intramuscularly via a codon-optimized plasmid administered in trans alongside CpG-optimized antigen-encoding plasmids. When co-administered with CpG-enriched mE6/HSP70 and mE7/HSP70 plasmids, IL-28B significantly enhanced antigen-specific CD8^+^ T cell responses and improved both prophylactic and therapeutic control of E6/E7-expressing tumors in mice. In side-by-side comparison, both IL-28B and IL-15 significantly enhanced CTL responses. However, IL-28B induced superior CTL responses and significantly higher granzyme B mRNA levels, suggesting more robust activation of cytotoxic effector pathways (61). These studies demonstrate the potential of IL-28B as a robust cytokine adjuvant capable of enhancing cellular immunogenicity in various animal models, suggesting broad utility for improving vaccine potency.

### GM-CSF

Granulocyte-macrophage colony-stimulating factor (GM-CSF), also known as colony-stimulating factor 2 (CSF2), is a monomeric glycoprotein secreted by macrophages, T cells, mast cells, natural killer cells, endothelial cells, and fibroblasts that functions as a pleiotropic cytokine that drives differentiation of myeloid precursors into APCs, enhances DC maturation, and supports the generation of T_H_1-biased immune responses. GM-CSF recruits and activates DCs and other myeloid cells at the site of antigen delivery. When co-delivered with vaccines, GM-CSF enhances antigen presentation and promotes robust humoral and cellular immune responses (114).

GM-CSF has been explored as an adjuvant since the early 1990s, with a 1994 review highlighting its potential to enhance immune responses by promoting DC maturation and increasing antibody titers in both animal and human studies (115). The cytokine was first used as a plasmid-encoded adjuvant in 1997, with co-delivery resulting in the enhancement of HIV-1 gag, pol, and env-specific antibody responses (81). GM-CSF has shown notable adjuvant capability in DNA vaccines targeting various infectious diseases (114). Plasmid-encoded GM-CSF has previously reported as well-tolerated in clinical trials evaluating DNA vaccines for advanced melanoma and prostate cancer (116–118).

Plasmid-encoded GM-CSF (pGM-CSF) enhanced the immunogenicity of an RBD-based DNA vaccine against SARS-CoV-2 by boosting both humoral and cellular responses, including robust neutralizing antibody production against ancestral and Omicron variants (66). pGM-CSF also promoted antigen expression, immune cell recruitment, germinal center B cell responses, and the formation of central and tissue-resident memory T cells, suggesting a multifaceted adjuvant role.

Likewise, mRNA-LNP delivery of a fusion gene of GM-CSF and IL-4 (GIFT4) broadly and robustly enhanced adaptive immune responses to influenza antigens in mice, including robust germinal center formation and lung-resident T cell induction (58). GM-CSF, as part of GIFT4, also promoted early germinal center formation and B cell activation in draining lymph nodes. Intradermal administration of this construct produced notable lung-resident T cell populations, indicating mucosal immune enhancement by GM-CSF-based adjuvanticity.

GM-CSF as a gene adjuvant enhances humoral responses and promotes DC activation, but its efficacy in boosting cellular immunity and protection varies widely by context, antigen, and delivery method. Encoded or locally secreted forms, especially via plasmid or mRNA, outperform recombinant cytokine delivery, with spatial-temporal control proving critical. Fusion constructs like GIFT4 further support the use of GM-CSF as a gene adjuvant, particularly for inducing germinal center and tissue-resident T cell responses. Overall, plasmid-encoded GM-CSF continues to be a promising adjuvant in nucleic acid vaccine development, with ongoing research focusing on optimizing its delivery and combination with other immunostimulatory agents to maximize vaccine efficacy.

## Chemokine adjuvants

Chemokines are a specialized class of cytokines that control chemotaxis through chemical signaling, often directing the migration of immune cells to sites of infection or vaccination. They are typically thought of in terms of ligands for specific receptors. Chemokine adjuvants are often noted for their ability to tailor vaccine-induced immunity in mucosal and peripheral contexts (**Table 1**).

### CCR10 ligands

Chemokine receptor 10 (CCR10) is expressed on IgA+ B cells and T cells at barrier surfaces, including the respiratory, gastrointestinal, and skin surfaces. CCR10 has two known ligands: chemokine ligands 27 (CCL27) and 28 (CCL28). We have previously reported that co-delivery of CCR10 ligands in the context of gag/pol/Env DNA immunogens supports increased protection from SHIV vaginal challenge in non-human primates (17).

CCL27, also known as cutaneous T cell-attracting chemokine (CTACK), is canonically known as a director of T lymphocyte chemotaxis in the epidermis. The chemokine was first used as a vaccine adjuvant by Kutzler and colleagues in 2010 (16), where plasmid-encoded CTACK (pCTACK) was shown to enhance systemic and mucosal immune responses to DNA vaccines, including increased antigen-specific IgG and IgA levels, as well as elevated IFN-γ production from CD8 T cells This approach also provided protection in a lethal influenza challenge.

Additional studies have characterized on the ability of plasmid-encoded CTACK to bolster mucosal immunity against respiratory pathogens like SARS-CoV-2 and influenza. We investigated the use of pCTACK co-delivered with a SARS-CoV-2 DNA vaccine in mice (67). Co-delivery of CTACK in the periphery led to increased spike-specific IgA at mucosal surfaces, but not in serum, suggesting a targeted mucosal response. pCTACK also led to higher frequencies of IFN-γ^+^ CD8^+^ T cells in the respiratory mucosa expressing a mucosal homing marker. pCTACK provided 100% protection against heterologous Delta variants in lethal challenge with complete survival, absence of weight loss, and reduced lung pathology compared to animals immunized with the spike DNA vaccine alone. When co-delivered with a DNA vaccine encoding a self-assembling influenza hemagglutinin (HA) head domain nanoparticle immunogen, pCTACK increased HA-specific antibody levels in the bronchoalveolar lavage and reduced lung pathology in a lethal challenge model relative to antigen alone (68).

Similarly, the second CCR10L, CCL28, also called mucosa-associated epithelial chemokine (MEC) has also been explored as a molecular adjuvant. We have previously reported that co-delivery of plasmid-encoded CCL28 (pMEC) with HIV-1 envelope DNA immunogens supported increased anti-HIV responses at mucosal sites including increased IgA in fecal extracts and frequencies of HIV-specific B cells in intestinal Peyer’s patches (13). These studies demonstrate that peripheral delivery of mucosal-homing chemokines can provide enhanced protection at key barrier sites of pathogen entry.

### CCR6 ligands

Chemokine Ligand 20 (CCL20), also known as liver activation regulated chemokine (LARC) and macrophage inflammatory protein-3 (MIP-3a) is strongly chemotactic cytokine for lymphocytes and weakly attracts neutrophils. MIP-3α is the only known natural ligand for CCR6 and plays a specialized role in targeting immature dendritic cells (iDCs). It has been explored as a gene-encoded adjuvant since at least 2014, where it was shown to direct antigen presentation toward CCR6^+^ iDCs, enhancing antigen uptake and priming adaptive responses (119–121). For instance, a 2014 study reported fusion of the malaria antigen to MIP-3α in a DNA vaccine, combined with the lipid-based adjuvant Vaxfectin, significantly improved protective efficacy in a murine challenge model, with MIP-3α enhancing targeting of antigen to immature dendritic cells, leading to sterilizing immunity comparable to that induced by irradiated sporozoites (119).

Subsequent studies have extended these observations. A 2020 report found that plasmid-encoded MIP-3α boosted immune responses when co-delivered with an HIV-1 gp140 DNA vaccine, followed by mucosal protein boosting (122). The chemokine increased antigen-specific antibodies in both serum and mucosal sites, including the vaginal vault and intestinal lumen, and promoted immune cell recruitment to mucosal tissues. More recently, in a 2024 SARS-CoV-2 DNA vaccine study, inclusion of MIP-3α enhanced both the magnitude and durability of antibody responses, with neutralizing titers sustained for at least 12 months after intramuscular (IM) electroporation (123). In parallel, intranasal delivery of the same plasmid (without encapsulation or electroporation) elicited significantly stronger lung-localized T-cell responses compared to controls. MIP-3α has also improved immunogenicity in murine melanoma DNA vaccine models (124–126).

Together, these results support MIP-3α (CCL20) as a broadly functional gene-encoded adjuvant. Co-delivery with plasmid vaccines enhances systemic and mucosal immunity, promoting both sustained antibody production and lymphocyte recruitment to barrier tissues. Across multiple platforms, including HIV-1 and SARS-CoV-2, MIP-3α has improved humoral and T-cell responses even in the absence of advanced delivery technologies, reinforcing its translational potential for vaccines requiring mucosal or long-term protection.

### CCR7 ligands

CCL19 and CCL21 are functional homologs and ligands for CCR7. They drive DC and T cell homing to lymph nodes, enhancing antigen presentation and T cell priming. CCL19 and CCL21 have been explored as vaccine adjuvants since the early 2000s, with many early studies in the cancer immunotherapy space. A 2004 study by Flanagan et al. used a recombinant vaccinia virus expressing CCL19 to induce a CD4^+^ T-cell dependent antitumor response in mice, marking one of its first vaccine applications (127).

Recent progress in plasmid-encoded CCL19 and CCL21 has been made in fish and mouse models. A 2020 study in flounder (*Paralichthys olivaceus*) reported bicistronic DNA plasmids encoding both the *Vibrio anguillarum* bacterial antigen (VAA) and either CCL3, CCL4, CCL19, or CCL21 (69). Co-immunization with CCL19 or CCL21 plasmids significantly enhanced protection in challenge, leading to a relative percent survival (RPS) of 78.38% and 72.97% respectively, compared to 40.54% with antigen alone. Co-expression of CCL19 also led to increased sIgM^+^, CD4-1^+^, and CD4-2^+^ lymphocyte populations and VAA-specific antibody levels.

CCL19a.2 is a teleost-specific functional homolog of mammalian CCL19 that retains core chemotactic and immunostimulatory functions. A DNA vaccine encoding both viral hemorrhagic septicemia virus (VHSV) glycoprotein and CCL19a.2 significantly elevated early expression of interferon- and cytokine-related genes in lymphoid tissues of zebrafish (70). While co-expression with CCL19a.2 did not significantly improve survival following viral challenge, it induced pronounced innate immune activation within the first two weeks post-immunization. It is important to note that potential differences in expression kinetics or cellular targets may influence CCL19 adjuvant effect across species (128). In a 2024 study, CCL19 used in an intranasal H7N9 HA DNA vaccine significantly enhanced both cellular and humoral immune responses in mice (71). When combined with polyethylene imine and chitosan for mucosal delivery, the vaccine induced strong local mucosal and systemic immunity, providing 100% protection against lethal virus challenge. Mice immunized with the CCL19 composite vaccine also exhibited increased levels of IL-2 and IFN-γ and robust IgA production.

These findings demonstrate the diverse potential of CCL19 and CCL21 as plasmid-encoded adjuvants, with demonstrated benefits to both systemic and mucosal immunity across different species. The ability of these chemokines to enhance antigen-specific responses and provide protection against pathogenic challenges positions them as promising adjuvants for use in nucleic acid vaccine design.

## Costimulatory and immunomodulatory adjuvants

Costimulation is one of the essential signals required for full T cell activation during adaptive immune responses. Costimulatory molecules are typically cell surface proteins, though some soluble or enzymatic immunomodulators can also enhance T cell activation through costimulatory-like effects. By providing secondary signals to T cells, they enhance their proliferation, survival, and differentiation following antigen recognition. Certain costimulatory ligands have been shown to act as adjuvants when co-delivered alongside gene-encoded immunogens (**Table 1**). Other immunomodulatory adjuvants for nucleic acid vaccines include enzymes such as adenosine deaminase (ADA), the complement fragment C3d, and lipid nanoparticle (LNP) delivery systems (**Table 2**).

### CD40L/CD154

CD40 ligand (CD40L or CD154) is a costimulatory molecule that enhances vaccine-induced immunity by promoting DC activation, B cell help, and germinal center formation via engagement of CD40 on APCs. CD40L has been explored as a vaccine adjuvant since the late 1990s, with early studies demonstrating its potential to enhance both humoral and cellular immune responses (136, 137). The first study using CD40L as a DNA plasmid-encoded adjuvant was in 2006, with duck CD154 enhancing specific antibody responses to hepatitis B virus (138).

In recent years, studies have shown the ability of CD40L to enhance both humoral and cellular immune responses across various disease models. A 2019 study (72) reported a DC-targeted CD40 DNA vaccine (DEC-CD40) suppressed Th17 cell responses and reduced kidney damage in a rat model of autoimmune glomerulonephritis.

A 2022 study reported fusing CD40L to the SARS-CoV-2 spike in a DNA vaccine enhanced immunogenicity and reduced lung pathology in Syrian hamsters post-challenge (73). CD40L acted as both a targeting ligand and intrinsic adjuvant, amplifying neutralizing antibody responses and improving protection. Returning to poultry vaccines, Huang et al. used Duck CD40L (dusCD40L) in the context of a DNA vaccine against Tembusu virus. CD40L co-delivery significantly boosted both humoral and cellular immune responses against Tembusu virus (74) resulting in significantly improved neutralizing antibody titers, IFNγ production, and viral clearance in challenge.

Kornuta et al. evaluated the use of a plasmid encoding soluble CD40L combined with the adjuvant Montanide™ GEL01 to enhance a DNA vaccine (pCIgD) targeting bovine herpesvirus 1 (BoHV-1) (75). The combination improved DC activation *in vitro* and significantly boosted humoral and cellular immune responses in vaccinated cattle, including increased virus-specific IgG subclasses, neutralizing antibodies, and IFNγ/IL-4 secretion. Upon viral challenge, animals receiving the CD40L-enhanced vaccine showed reduced clinical symptoms, lower viral shedding, and stronger proliferation of lymphocytes, indicating improved protective efficacy.

These studies highlight the multi-faceted impact of CD40L as an adjuvant, capable of enhancing both humoral and cellular immune responses across animal models. CD40L was observed to mitigate immunopathology and facilitate antigen targeting to DCs, suggesting its potential as a molecular adjuvant for infectious disease and autoimmune vaccine strategies.

### CD80/CD86

CD80 and CD86 (B7-1/B7-2) are functional homologs that serve as ligands for the co-stimulatory receptor CD28, which is constitutively expressed on naïve T cells. These molecules are expressed on APCs, with DCs typically expressing both, while monocytes and macrophages predominantly express CD86. Engagement of CD28 by CD80/86 delivers the secondary activation signal required for T cell activation, promoting proliferation, IL-2 production, resistance to anergy, and expression of the anti-apoptotic protein Bcl-xL (76, 139–141).

Following identification in the early 1990s (139, 140), CD80/86 were first used as DNA-encoded adjuvants in 1997, recognized for their ability to induce cellular responses. Co-immunization with CD86, and not CD80, was found to significantly enhance specific T-cell mediated responses, highlighting CD86’s more potent effect as a molecular adjuvant (141, 142).

In 2022, Liu et al. identified a homolog of CD80/86 expressed primarily on APCs in flounder and constructed a bicistronic DNA vaccine co-expressing CD80/86 with the *Vibrio anguillarum* antigen OmpK (76). Co-expression of CD80/86 enhanced humoral immune responses, evidenced by increased IgM+ and CD4+ cell proportions and elevated expression of activation markers and cytokines at the injection site. The vaccine combining CD80/86 with OmpK significantly improved survival after bacterial challenge compared to antigen alone, supporting the adjuvant potential of CD80/86 in teleost fish. Phylogenetic analysis revealed that bony fish CD80/86 is more closely related to mammalian CD86 than to CD80. CD80/86 has also been implemented as a DNA-encoded adjuvant in several cancer vaccines (143).

Despite early promise in the 2000s, interest in CD80/CD86 as molecular adjuvants has declined. Over time, research has shifted toward stronger or more multifunctional co-stimulatory ligands, like CD40L, which not only activate T cells but also directly license DCs and promote cytokine production.

### Adenosine deaminase

Adenosine deaminase-1 (ADA) is a highly-conserved enzyme, known as a key regulator of the immune system and purine metabolism. ADA regulates intra- and extracellular levels of adenosine and has both enzymatic and extraenzymatic immune functions. Pegylated bovine ADA-1 (PEGademase) has been FDA-approved for use in humans since 1990 as an enzyme replacement therapy (ERT) for ADA-deficient severe combined immunodeficiency (ADA-SCID). The established regulatory and safety record of PEGademase offers potential benefit for clinical development of plasmid-encoded ADA. In 2017, Tardif *et al*. identified *ADA1* expression as a key driver of T follicular helper (T_FH_) cell differentiation, both within germinal centers (GC T_FH_) and in circulating T_FH_ (cT_FH_) and demonstrated that recombinant ADA enhanced antibody production in *in vitro* T_FH_ and B cell coculture assays (144). This prompted the design of plasmid-encoded ADA-1 (pADA) for evaluation in the context of DNA immunization. In 2020, pADA was found to enhance the maturation of myeloid DCs and promote IL-6 secretion, fostering a microenvironment conducive to T_FH_ polarization (9). Co-administration of pADA with an HIV-1 envelope DNA vaccine significantly increased T_FH_ cell frequencies in draining lymph nodes and boosted serum HIV-specific IgG responses (9). In these studies, when delivered alongside both DNA and protein immunogens, pADA uniquely enabled the induction of homologous HIV-1 neutralizing antibodies, highlighting its capacity to qualitatively enhance germinal center and antibody responses *in vivo*.

Recent findings have further characterized these effects. In 2023, scRNAseq analysis revealed that aged mouse T cells had decreased *ADA1* transcripts and we hypothesized that pADA could enhance vaccine-induced immunity in aged models of SARS-CoV-2 immunization and challenge. Indeed, we demonstrated that co-delivery of pADA significantly enhances both cellular and humoral responses in aged mouse models (7). Co-delivery of ADA enhanced both cellular and humoral responses to a SARS-CoV-2 DNA vaccine in aged mice, ameliorating age-associated declines in IFNγ secretion and antibody quality (7). pADA broadened the affinity and breadth of spike-specific antibodies and promoted a T_H_1-skewed transcriptional profile in lymph node lymphocytes while reducing FoxP3 expression. These effects correlated with reduced viral load and improved survival following SARS-CoV-2 challenge, demonstrating that pADA restores vaccine efficacy in immunosenescent hosts. Together, these studies highlight pADA as a broadly-active molecular adjuvant that enhances cellular and humoral immunity by shaping T and B cell interactions, promoting dendritic cell activation, and mitigating age-associated declines in immune responsiveness.

### C3d

C3d, a fragment of the complement component C3, binds to complement receptor 2 (CR2) which is located on the surface of follicular dendritic cells (FDC), B cells, and T cells. C3d was first used as an adjuvant in 1996, with 2 or 3 copies of recombinant C3d delivered alongside the model antigen HEL, drastically increasing antibody responses by up to 10,000-fold (145). This finding highlighted the ability of C3d to amplify immune responses by targeting CR2/CD21 and lowering the activation threshold for B cell responses.

Recent studies have investigated the impact of C3d fusion across the pDNA and mRNA platforms. A 2019 study in pigs, reported a DNA vaccine encoding a fusion of porcine C3d and the Porcine Circovirus Type 2 (PCV2) ORF2 protein induced cross-protective immunity against different PCV2 genotypes (77). The C3d-fused construct (pVOC3) contained three copies of C3d and elicited stronger PCV2-specific antibody responses, increased IFNγ-secreting T cells, and reduced viremia compared to the non-adjuvanted construct (pVO).

A 2023 study investigated various improvements to mRNA vaccines including C3d fusion to the spike or RBD antigens (78). C3d fusion significantly enhanced immunogenicity, inducing up to tenfold higher antibody titers in mice compared to unmodified antigen mRNA. The C3d fusion promoted both humoral and cellular immune responses, with evidence of balanced Th1/Th2 polarization influenced by LNP composition and delivery route. This strategy also avoided systemic inflammation and showed efficacy against SARS-CoV-2 variants. These studies demonstrate the potential of C3d as a molecular adjuvant for nucleic acid vaccines, particularly for enhancing humoral immunity.

### Adjuvanticity of lipid delivery

The first ionizable lipids were developed for DNA transfection starting in 1989, followed by iterations developed for siRNA delivery through the 2000s, and more recent formulations optimized for mRNA constructs (40). The development of lipid nanoparticles (LNPs) to deliver mRNA vaccine antigens was necessitated by the inherent instability of mRNA; LNPs protect the mRNA cargo and promote cellular uptake. Recently, multiple studies have noted and described an inherent adjuvanticity of LNPs (146–149) and systems vaccinology approaches have characterized innate and adaptive immune responses to mRNA-LNP (150–152). In 2021, Alameh et al. demonstrated that lipid nanoparticles possess intrinsic adjuvant activity independent of their mRNA cargo, eliciting strong T follicular helper cell responses and durable antibody production (147). This immunostimulatory effect was dependent on IL-6 induction but occurred independently of MyD88 or MAVS signaling. When co-administered with recombinant protein antigens, empty LNPs elicited superior humoral responses compared to a benchmark squalene adjuvant, underscoring their potential as stand-alone adjuvants in vaccine formulations.

Ndeupen et al. confirmed the highly inflammatory properties of LNP’s, noting empty LNP’s activate multiple inflammatory pathways, induce production of IL-1β and IL-6, and trigger *l1b* and *Nlrp3* -associated inflammasome activation (146). Tahtinen et al., noted that RNA-LNP vaccines, regardless of administration route, activate the IL-1–IL-1ra axis and drive systemic inflammation in humans through IL-1β-dependent cytokine cascades, including IL-6 (152). In 2025, LNPs were identified to activate NF-κB and IRF signaling pathways in monocytes via Toll-like receptor 4 (TLR4) in the absence of mRNA and further confirmed as the primary driver of innate immune activation using knockout cell lines (153). These findings characterize the immunostimulatory effects of LNP-mediated delivery, representing a tunable adjuvant component and potent construct design capability. While reducing reactogenicity has been an area of focus in the RNA space, the intrinsic adjuvant properties of LNP formulations offer a substantive and promising route to boost immunogenicity for DNA constructs.

Recently, ionizable LNP-encapsulated plasmid DNA (DNA-LNPs) have emerged as an immunogenic vaccine modality against infectious diseases (36–38, 129, 130, 132, 133, 154) and cancer (131, 134, 135). Numerous studies (**Table 2**) have characterized different ionizable lipids, namely SM-102, ALC-0315, MC3, and KC2. The relationship between the ionizable lipid amine groups and DNA backbone phosphates, or N/P ratio, has also been examined. Various studies report an N/P ratio of approximately 6 (or lower lipid to DNA weight ratios) (36, 37, 129, 134, 154). This variable was studied in depth with an H1N1 HA-expressing DNA-LNP, where Tursi et al. report that higher N/P ratios led to improved biophysical characteristics such as particle size and zeta potential, supporting improved immunogenicity (38). The LNP component, specifically the ionizable lipid, has intrinsic adjuvanticity as previously described. Unlike mRNA-LNPs, DNA-LNP formulations additionally drive cGAS-STING signaling due to the presence of plasmid DNA in the cytoplasm; this pathway contributes to the activation of innate immune subsets associated with immunization (38). Beyond the intrinsic adjuvanticity of ionizable lipids, molecular adjuvants have also been evaluated in combination with DNA-LNP vaccines, including studies utilizing CD40L and OX-40L (134).

**Table 2:** Adjuvanticity of lipid delivery.

Antigen	Ionizable Lipid, N/P or weight ratio (if specified)	Significant Findings	Reference
**SARS-CoV-2 spike (wild-type and Omicron BA.1)**	SM-102 (N/P Ratio = 6)	Induced comparable or superior humoral immunity to a matched mRNA-LNP in multiple rodent models, improved protection from challenge	Liao et al. Molecular Therapy Methods & Clinical Development (36)
**SARS-CoV-2 spike (Delta variant) fused to CD40L ectodomain**	KC2 and SM-102 (N/P Ratio = 6)	Relative to naked DNA, LNP formulation led to superior neutralization titers and reduced viral loads in challenge, was dose-sparing in hamsters	Tamming et al. Molecular Therapy Methods & Clinical Development. (129)
**Influenza H3N2 HA**	MC3 (N/P ratio = 4.5)	Induced antibody titers and T cell responses in swine, with significantly reduced viral shedding and lung pathology in challenge	Nguyen et al. mSphere. (130)
**Influenza H1N1 HA**	MC3 (N/P ratio = 4.5 and 5.5)	Induced humoral and cellular responses as well as mediated protection in an influenza challenge model in mice and swine	Nguyen et al. mSphere. (130)
**HPV16 and HPV18 E6/E7**	MC3, SM-102, and ALC-0315	Enhanced T Cell responses relative to DNA delivered using electroporation. SM-102-based formulations drove superior immunogenicity relative to MC3 and ALC-0315.	Li et al. Vaccines. (131)
**SARS-CoV-2 spike (Gamma variant)**	Ionizable lipid not specified (Lipid to DNA weight ratio = 10:1)	Induced robust humoral and cellular immune responses, reduction in viral load, lung pathology in SARS-CoV-2 challenge models in mice and hamsters	Guimaraes et al. Nature Communications (37)
**Influenza H1N1 HA and SARS-CoV-2 spike (wild-type)**	SM-102 (N/P ratios 10.5, 5.3, and 2.6)	Robust innate immune responses, notably migratory DCs. Comparable humoral immune responses and superior T cell responses to mRNA-LNP and adjuvanted protein. Protection from challenge in SARS-CoV-2 model	Tursi et al. Cell Rep Med. (38)
**SARS-CoV-2 spike (Omicron variant)**	SM-102 (Total lipid to DNA weight ratio = 20:1)	Induced humoral immune responses and is protective in a wild-type SARS-CoV-2 challenge model in hamsters	Yang et al. Molecular Therapy Nucleic Acids. (132)
**B. burgdorferi OspC**	KC2	Elicited binding and functional antibody responses, mediates protection in B. burgdorferi challenge	Pfeifle et al. Frontiers in Immunology. (133)
**OX-40L**	KC2, MC3, C12-200 (Ionizable lipid to DNA weight ratio 5:1)	Intratumoral delivery of plasmid DNA in LNPs led to a reduction in tumor burden. OX-40L-expressing plasmid in combination with an siRNA led to improved challenge outcomes.	Qin et al. Journal of Controlled Release. (134)
**SARS-CoV-2 spike, PD-L1, p53^R172H^**	SM-102, ALC-0315, MC3	Induced superior humoral and cellular immune responses relative to electroporation. Expression of PD-L1/p53 variant led to humoral immune responses and a reduction in tumor burden	Chai et al. Molecular Cancer. (135)

## Remaining challenges

Despite promising advances, gene-encoded adjuvants face several remaining limitations that constrain their translation and optimization. Beyond IL-12, few molecular adjuvants have been clinically evaluated in infectious disease contexts (82–85). Regulatory frameworks specific to gene-encoded adjuvants, particularly for mRNA platforms encoding cytokines or co-stimulatory ligands, are underdeveloped. Notably, most clinical evaluation of gene-encoded adjuvants has occurred in oncology, across both the DNA and mRNA platforms (31, 32, 48, 49).

A core technical challenge is achieving precise control over expression kinetics. Unlike conventional adjuvants with defined pharmacokinetics, gene-based adjuvants rely on *in vivo* transcription and translation, which introduces variability in expression timing, intensity, and tissue distribution. Because plasmids can persist in host cells for extended periods, sustained antigen or cytokine expression raises concerns about immune tolerance (155). Several studies report that prolonged or dysregulated expression can impair adaptive immunity, promote T-cell exhaustion, or diminish vaccine efficacy (28). Despite advances in vector design and delivery strategies, achieving real-time, tunable expression control *in vivo* remains difficult, particularly in balancing immunogenic potency with safety in dynamic immune environments.

While sustained expression may support effector T-cell persistence, it also carries risks. In prophylactic settings, prolonged antigen exposure can impair central memory formation or trigger tolerance and anergy. In chronic infection models such as hepatitis B virus (HBV), persistent antigen expression from DNA vaccination has led to circulating immune complexes and tissue pathology, raising context-dependent safety concerns (Hanke, 2006). These findings emphasize that unregulated or extended expression may compromise vaccine performance depending on disease setting and immunological mechanism (156).

Temporal control is also critical for optimizing immune outcomes. Irvine et al. (157) showed that the timing of cytokine expression from gene-encoded adjuvants affects cytokine expansion and shifts T helper polarization. For example, GM-CSF administered before versus after immunization produced divergent Th1/Th2 responses. While concerns persist regarding chronic inflammation from sustained proinflammatory cytokine expression (157), preclinical animal studies suggest that local adjuvant production is self-limiting, inducing effects in local draining lymph nodes and at the site of injection while being undetectable in circulation.

Another limitation is incomplete mechanistic understanding for many molecular adjuvants. While cytokines and co-stimulatory ligands have well-defined immunological functions, their specific roles when encoded as nucleic acid adjuvants remain incompletely characterized. Molecules such as ADA and C3d have demonstrated consistent immune enhancement across studies, yet their mechanisms of action are still not fully resolved.

Finally, safety remains a concern, particularly the risk of overactivation. Cytokines like IL-2 and IL-12 showed significant toxicity when delivered as recombinant proteins, although plasmid-encoded delivery and localized expression has significantly mitigated reactogenicity. For DNA-based platforms, a monitored safety risk is genomic integration. FDA guidance requires that integration frequencies remain below the spontaneous mutation rate. Existing studies overwhelmingly support the safety of DNA vaccines, with the approval of ZyCoV-D in 2021 representing a milestone in regulatory acceptance (26, 158).

## Discussion and concluding remarks

Adjuvant development has historically been primarily empirical, with mechanistic insight often applied retrospectively. However, evolving methodologies, including growing insight into platform-specific immune signatures, supports a continued shift toward rational construct design. DNA and RNA vaccines differ in antigen expression kinetics and innate sensor engagement, shaping their downstream immune profiles (159, 160). mRNA–LNP vaccines activate endosomal and cytosolic RNA sensors such as TLR7/8 and RIG-I, often driving strong CD4 and antibody responses (161–163). Their rapid and transient cytosolic expression is frequently associated with high reactogenicity. In contrast, DNA vaccines often engage sensors including cGAS–STING and TLR9 and tend to induce more delayed, sustained antigen expression, eliciting characteristically strong CD8 T cell responses (27, 28, 38). These general trends point to opportunities for tuning adjuvant strategies to better complement each platform. Mechanistically informed adjuvants offer potential to tailor innate activation and moderate reactogenicity. Aligning adjuvants with the immune kinetics and qualitative response patterns of each platform may improve both efficacy and tolerability in next-generation vaccine development.

Route of administration and target tissue influence antigen presentation, innate activation, and immune priming, making them important variables in adjuvant design. Intramuscular (IM) injection remains the standard for nucleic acid vaccines due to practical advantages and regulatory precedent, but skeletal muscle contains few resident antigen-presenting cells (APCs), often requiring adjuvants that promote APC recruitment or strong immunostimulatory formulations like lipid nanoparticles. In contrast, intradermal and mucosal routes target tissues rich in specialized APCs and may support efficient priming at lower doses. However, these routes also introduce challenges, including local inflammation or tolerance induction, that necessitate route-specific tuning of adjuvant potency and formulation.

DNA vaccines have demonstrated greater versatility in non-intramuscular delivery routes (28, 29), including intradermal administration (164, 165), chitosan-based formulations, electroporation, and mucosal-targeting adjuvants. In contrast, mRNA–LNP vaccines are predominantly administered intramuscularly, where they have shown robust immunogenicity, with most innovation focused on particle stabilization, such as PEGylation, or optimizing lipid composition to enhance delivery and reduce reactogenicity (166). For both platforms, adaptation strategies include chemokines like CCL20 to recruit mucosal dendritic cells (167–170), mucoadhesive or pH-sensitive carriers for stability (168–170), and tissue-matched PRR agonists (171, 172). Some adjuvants show route-dependent efficacy. For example, CpG performs best parenterally (173, 174), while cholera toxin derivatives show greater efficacy at mucosal surfaces (175, 176). Ongoing work explores candidates like chitosan and cGAMP to improve mucosal delivery without excessive inflammation (28, 29). As rational adjuvant design advances, aligning formulations with the immunological features of each delivery route will be key to improving vaccine performance.

Nucleic acid vaccines have made remarkable progress since their inception over three decades ago. Molecular adjuvant technology has developed in parallel with nucleic acid vaccine platforms, with recent studies refining their characterization across diverse classes of antigens and building on the strong foundation of adjuvant delivery. Molecules such as IL-12, IL-2, and GM-CSF have shown continued promise in plasmid-encoded formats, where localized delivery has been essential for mitigating safety concerns. In contrast, candidates like IL-15, IL-18, and CD80/CD86 have faced developmental setbacks despite encouraging early data. Emerging adjuvants such as adenosine deaminase reflect the expanding platform-specific toolkit. Although challenges remain, including the need for precise expression control and a limited clinical footprint, momentum continues to build for DNA and mRNA vaccine technologies.

Molecular adjuvants are becoming increasingly mechanistically tailored and platform-adapted, solidifying their central role in nucleic acid vaccine technology. The distinct immunological profiles elicited by DNA and RNA vaccines demand adjuvants that are matched to their kinetics, antigen presentation pathways, and reactogenicity. Additionally, state-of-the-art computational tools (177) combined with structure-guided methods enable the development of a new generation of adjuvant molecules designed *de novo* (178–181). Advances in vector engineering, delivery technologies, combination approaches, optimization of immunogen–adjuvant pairings, and increased assessment in human patients will enable continued development for nucleic acid vaccines, supporting broader platform adoption to address major global health challenges.
